# A Genome-Wide Screen in *Saccharomyces cerevisiae* Reveals a Critical Role for Oxidative Phosphorylation in Cellular Tolerance to Lithium Hexafluorophosphate

**DOI:** 10.3390/cells10040888

**Published:** 2021-04-13

**Authors:** Xuejiao Jin, Jie Zhang, Tingting An, Huihui Zhao, Wenhao Fu, Danqi Li, Shenkui Liu, Xiuling Cao, Beidong Liu

**Affiliations:** 1 State Key Laboratory of Subtropical Silviculture, School of Forestry and Biotechnology, Zhejiang A&F University, Lin’an, Hangzhou 311300, China; jinxuejiao1991@cau.edu.cn (X.J.); zhangjie@stu.zafu.edu.cn (J.Z.); antingting@stu.zafu.edu.cn (T.A.); zhaohuihui@stu.zafu.edu.cn (H.Z.); fuwenhao@stu.zafu.edu.cn (W.F.); lidanqi@stu.zafu.edu.cn (D.L.); shenkuiliu@nefu.edu.cn (S.L.); 2 Department of Chemistry and Molecular Biology, University of Gothenburg, Medicinaregatan 9C, SE-413 90 Goteborg, Sweden; 3 Center for Large-Scale Cell-Based Screening, Faculty of Science, University of Gothenburg, Medicinaregatan 9C, SE-413 90 Goteborg, Sweden

**Keywords:** LiPF_6_, genome-wide screen, mitochondrial damage, ROS, ATP content, oxidative phosphorylation

## Abstract

Lithium hexafluorophosphate (LiPF_6_) is one of the leading electrolytes in lithium-ion batteries, and its usage has increased tremendously in the past few years. Little is known, however, about its potential environmental and biological impacts. In order to improve our understanding of the cytotoxicity of LiPF_6_ and the specific cellular response mechanisms to it, we performed a genome-wide screen using a yeast (*Saccharomyces cerevisiae*) deletion mutant collection and identified 75 gene deletion mutants that showed LiPF_6_ sensitivity. Among these, genes associated with mitochondria showed the most enrichment. We also found that LiPF_6_ is more toxic to yeast than lithium chloride (LiCl) or sodium hexafluorophosphate (NaPF_6_). Physiological analysis showed that a high concentration of LiPF_6_ caused mitochondrial damage, reactive oxygen species (ROS) accumulation, and ATP content changes. Compared with the results of previous genome-wide screening for LiCl-sensitive mutants, we found that oxidative phosphorylation-related mutants were specifically hypersensitive to LiPF_6_. In these deletion mutants, LiPF_6_ treatment resulted in higher ROS production and reduced ATP levels, suggesting that oxidative phosphorylation-related genes were important for counteracting LiPF_6_-induced toxicity. Taken together, our results identified genes specifically involved in LiPF_6_-modulated toxicity, and demonstrated that oxidative stress and ATP imbalance maybe the driving factors in governing LiPF_6_-induced toxicity.

## 1. Introduction

Lithium-ion batteries are widely used, worldwide, in the field of electronic and electrical appliances, especially in new energy vehicles. As a result, demand and production of lithium-ion batteries has continued to grow rapidly in recent years [1,2,3]. With the disposal of spent lithium-ion batteries, increased concentrations of lithium-containing compounds are entering the environment, resulting in a potential contamination and threat to all types of organisms, including animals, plants, and microbes. Lithium hexafluorophosphate (LiPF_6_) is one of the leading electrolytes in lithium-ion batteries [4]. It can undergo chemical reactions, such as hydrolysis, decomposition, and combustion, to produce fluorine- and lithium-containing compounds, and might lead to fluorine and lithium toxicity [5].

Lithium is not an essential element for life and it usually occurs in soil and water at a low concentration. Low levels of lithium have many beneficial effects on living organisms, such as DNA synthesis and repair in microbes [6], plant growth stimulation [7], and life span extension in *Drosophila* [8]. Lithium has also been a pharmacological therapeutic option for bipolar disorder [9]. However, high levels of lithium are toxic, as evidenced by the induction of necrotic lesions in plants [10], and various acute and chronic responses in humans and animals [11]. To date, studies regarding the underlying mechanism of lithium toxicity have mainly suggested an association with oxidative stress and ion homeostasis disruption [11]. High concentrations of lithium have been reported to induce high reactive oxygen species (ROS) formation and to reduce mitochondrial membrane potential, which together contribute to limited energy production and lipid peroxidation [12,13,14]. Additionally, lithium can replace other cations (Na^+^, K^+^, Ca^+^), and specifically competes with magnesium ions (Mg^2+^), thus interrupting ion channel activity, Na^+^/K^+^ homeostasis, and the activity of magnesium-containing enzymes [15,16,17]. Other studies have indicated that lithium in high concentrations alters nucleic acid and protein biosynthesis [17], as well as introduces endoplasmic reticulum stress and N-glycan modification in certain conditions [18]. Likewise, fluorine is also toxic to cells, and hexafluorophosphates quaternary ammonium salts has been reported to lead to oxidative stress [19]. Fluorine can also induce cell apoptosis [20] and is toxic to the central nervous system, affecting neuron cell activity and ion transport [21].

Although some basic mechanisms of lithium toxicity have been revealed, the lithium compounds used in most studies are lithium chloride (LiCl), lithium hydroxide (LiOH), and lithium carbonate (Li_2_CO_3_) [5,7,13,22,23,24]. In contrast, limited information is available regarding the cytotoxicity mechanism of hexafluorophosphate or the comprehensive impacts of fluorine and lithium caused by contamination from lithium-ion batteries. It is also not clear whether similar mechanisms are utilized by cells to protect themselves from these toxins, compared with the more-studied lithium-containing compounds. Thus, it is important to investigate LiPF_6_ specifically, as a representative stressor, to characterize the mechanism of toxicity of lithium-ion battery contamination and the specific responses of cells to it.

The yeast model has been used extensively to study the toxicity and targets of various chemicals and drugs, due to the genetic similarity of yeast to other eukaryotes, and the diverse yeast collections available to researchers [25,26,27,28]. In previous work, a genome-scale genetic screen of the yeast non-essential gene deletion library identified 114 LiCl-sensitive and 6 LiCl-tolerant mutations [29]. In the present study, we first evaluated and compared the cytotoxicity of LiPF_6_, LiCl, and NaPF_6_ in *Saccharomyces cerevisiae*, and confirmed that the cytotoxicity of LiPF_6_ is different from that of LiCl and NaPF_6_ at the same concentration. High concentrations of LiPF_6_ can induce mitochondrial dysfunction, oxidative stress, and lowered ATP yield. Then, using the yeast deletion collection [30,31], a genome-wide screen was performed to identify the specific genes involved in LiPF_6_-mediated cytotoxicity. We identified 75 genes that may contribute to LiPF_6_ tolerance. These genes had not been previously reported to modulate LiPF_6_ resistance, and only a few of them were found to overlap with previously identified LiCl-sensitive genes [29]. This implies the existence of specific toxicity of LiPF_6_ and specific cellular responses to it. In further research, we found that oxidative phosphorylation-related genes are required for tolerance to LiPF_6_ and counteraction of LiPF_6_-induced ROS accumulation. Deletion of these genes also reduced ATP yield under LiPF_6_ treatment. Not only did our study reveal that the toxicity of LiPF_6_ is not a simple superposition of two ion poisons (Li^+^ and PF_6_^−^), but also elucidated the processes by which LiPF_6_ induces cytotoxicity and the cellular responses to LiPF_6_.

## 2. Materials and Methods

### 2.1. Growth Curve Measurement

BY4741was cultivated in yeast peptone dextrose (YPD) medium (1% yeast extract, 2% peptone, and 2% glucose) overnight and then diluted to an OD_600_ of 0.1 in YPD supplemented with 0 mM, 1 mM, 2 mM, 3 mM, 4 mM, and 5 mM of LiPF_6_ (dissolved in ddH_2_O, L822100, Macklin, Shanghai, CN), LiCl (dissolved in ddH_2_O, L9650, Sigma-Aldrich, St. Louis, MO, USA), NaPF_6_ (dissolved in ddH_2_O, 208051, Sigma-Aldrich, St. Louis, MO, USA), or both LiCl and NaPF_6_. The cell density of the cultures was determined, using a spectrophotometer (Ultrospec 2100 Pro, Biochrom, St. Albans, UK), at different time points from 0 to 24 h. The growth curve of BY4741 in yeast peptone glycerol (YPG) medium (1% yeast extract, 2% peptone, and 3% glycerol) with 0 mM or 4 mM LiPF_6_ was measured from 0 to 144 h, as described above.

### 2.2. Inhibition Zone Experiment

The toxicity of LiPF_6_, LiCl, and NaPF_6_ to yeast was evaluated through the filter diffusion method. The yeast strain was first spread on solid media. Meanwhile, the filters were soaked in different concentrations of three compounds, respectively. Then, the filters were placed on the culture dishes coated with the yeast and plates were cultured at 30 °C for 24 h for the detection of the inhibition zone. The inhibition zone of each sample was measured using a ruler and photographs of the different plates were taken.

### 2.3. Genome-Wide LiPF_6_ Screen

The collection of nonessential haploid MATa deletion strains used to make the synthetic genetic array (SGA) collection was derived from BY4741, and was a gift provided by Prof. Charles Boone (Toronto University, Toronto, ON, Canada) [31,32]. The strains in the collection were arrayed in the 384-format. Firstly, the strains, in 384-well frozen stock plates, were spotted onto YPD agar plates (with G418 added) using 384-pining replicators operated by a Singer Rotor (Singer Instruments, Somerset, UK) and the cells were incubated at 30 °C. Then, each 384-arrayed mutant group was replicated in quadruplicate to yield four identical arrays to eliminate operational differences, each with 384 mutant strains, plated on a single plate containing either no LiPF_6_ or 3 mM LiPF_6_ to generate a 1536-density array. These array plates were incubated for 2 days at 30 °C. Images of the plates were taken with Phenobooth (Singer Instruments, Somerset, UK) and growth assessment comparing the growth of individual mutants with or without LiPF_6_ was performed using SGAtools, as described previously [33]. SGAtools. Available online: http://sgatools.ccbr.utoronto.ca/ (accessed on 13 April 2021). Briefly, high-quality images of the plates were first analyzed by SGAtools to produce raw colony size measurements, and then three important biases in colony size measurements were corrected, including plate effect, row/column effect, and spatial effect. The surrounding strains on each plate array are the control strains for biases correction. Fitness scores were calculated against the control experiment by quantifying the deviation from the expected fitness. Mutants that were sensitive to LiPF_6_ were selected with a cut-off of less than −0.2, since scores below −0.2 usually indicate a relatively strong effect and based on our previous study most of the hits with a score less than −0.2 can be confirmed by other methods [34]. The experiments were repeated three times and the scores of each mutant in three independent experiments are shown in Table 1.

### 2.4. Functional Enrichment and Interaction Network Analysis

Enrichment of Gene Ontology (GO) terms in the gene sets of interest was analyzed using the GO Term Finder in the Saccharomyces Genome Database [35]. GO Term Finder. Available online: https://www.yeastgenome.org/goTermFinder (accessed on April 13th, 2021). Enrichment of Kyoto Encyclopedia of Genes and Genomes (KEGG) was analyzed using KOBAS [36]. KOBAS. Available online: http://kobas.cbi.pku.edu.cn/kobas3 (accessed on April 13th, 2021). The background in GO and KEGG enrichment analysis was the list of genes for screening. The functional classification analysis was based on the functional description from the Saccharomyces Genome Database. Saccharomyces Genome Database. Available online: https://www.yeastgenome.org/ (accessed on April 13th, 2021). GeneMANIA was used to analyze the co-localizations and genetic and physical interactions of the LiPF_6_-sensitive mutants [37]. GeneMANIA. Available online: genemania.org (accessed on April 13th, 2021).

### 2.5. Complementation Strain Construction and Spot Tests

The plasmids for the complementation assays were extracted from Molecular Barcoded Yeast ORF 1.0 (MoBY ORF 1.0 Library) [38], a gift provided by Prof. Charles Boone (Toronto University, Toronto, ON, Canada). These plasmids all express the URA3 gene, which can be used as a selectable marker. The extracted plasmids and empty vectors were transformed into corresponding deletion mutant strains and transformants were selected on SD-Ura agar plates. The complementation strains were confirmed by PCR with the primers listed in Table S1. For the spot test assays, overnight cultures of different strains were adjusted to an OD_600_ of 0.1 and cultured at 30 °C to reach the mid-log phase. Then, cultures were serially diluted and spotted on plates with or without LiPF_6_. After incubation at 30 °C, yeast growth was observed.

### 2.6. Mitochondrial Morphology Observation

Mitochondrial morphology was observed in wild-type cells expressing the mitochondrial matrix protein Ilv3-GFP or by MitoTracker Red CMXRos (M-7512, ThermoFisher Scientific, Waltham, MA, USA) staining. A strain containing Ilv3-GFP was picked up from the commercial Yeast GFP Clone Collection [39]. Yeast GFP Clone Collection. Available online: www.invitrogen.com/clones (accessed on April 13th, 2021). For staining, pre-cultures were diluted to an OD_600_ of 0.1 and then cultured in YPD medium with or without LiPF_6_. After 6 h culture, 1 OD cells were collected and resuspended in 200 μL fresh medium. MitoTracker Red CMXRos was added into cell cultures to a final concentration of 50 nM to stain mitochondria, and was allowed to sit at room temperature for 30 min. Then, cells were washed in phosphate buffered saline (PBS) two times before observation using a Zeiss Axio Observer 7 with Z stacks. The cells with tubular or fragmented mitochondria were counted and the proportion of these cells relative to the larger population of cells was calculated. To ensure the accuracy of the result, at least 200 cells were examined in each sample for each replicate.

### 2.7. ROS Measurement

ROS levels were measured as previously described [40,41]. Overnight cultures of wild-type and deletion mutant strains were diluted to an OD_600_ of 0.1 and cells were allowed to grow to reach mid-log phase in SD-His before being treated with 0 mM or 1 mM LiPF_6_ for 1 h at 30 °C. Then, cells were adjusted to an OD_600_ of 0.5, and 2 mL of the cultures were collected. Cells were washed twice with PBS, followed by incubation with 10 μM 2′,7′-dichlorofluorescein diacetate (H_2_DCF-DA) (S0033S, Beyotime, Shanghai, CN) in the dark for 30 min. Cells were washed in PBS two times and fluorescence was observed using a Zeiss Axio Observer 7 with GFP filter lens. For each strain, the proportion of H_2_DCF-DA-positive cells was calculated, and the fluorescence signal per cell was quantified using ImageJ software, as described previously [41,42]. Briefly, after background fluorescence was removed from the image, the total fluorescence of all the cells was measured and then divided by the number of cells.

### 2.8. Western Blot

Strains containing GFP-tagged ORF at the C-terminus end were picked up from the commercial Yeast GFP Clone Collection [39]. Yeast GFP Clone Collection. Available online: www.invitrogen.com/clones (accessed on April 13th, 2021). Overnight cultures were diluted to an OD_600_ of 0.1 and cells were allowed to grow to reach the mid-log phase in SD-His before being treated with 0 mM or 1 mM LiPF_6_ for 1 h at 30 °C. Then the cells were collected and treated with 0.2 M NaOH for 10 min at room temperature and boiled in HU buffer (200 mM phosphate buffer, pH 6.8, 8 M urea, 5% *w/v* SDS, 1 mM EDTA, 100 mM DTT, bromophenol blue) for 10 min. Denatured proteins were separated on a 10% SDS-PAGE gel and transferred to a nitrocellulose membrane. After sequential incubation with primary antibody and secondary antibody, signals were detected by using electrochemiluminescence (Amersham Imager 600, GE Healthcare Life Sciences, Boston, MA, USA). Antibodies to GFP (ab6556, Abcam, Cambridge, UK), Flag (14793S, Cell Signaling Technology, Danvers, MA, USA), and Pgk1 (ab113687, Abcam, Cambridge, UK) were used. Pgk1 served as the loading control and bands of Cox5a protein were quantified using ImageJ software.

### 2.9. Measurement of Mitochondrial ATP Synthesis

ATP synthesis based on isolated mitochondria was measured using a previously described method [43]. Briefly, cells were grown to saturation and diluted to an OD_600_ of 0.1 in YPD. Cells were grown to an OD_600_ of 0.5 before treated with 0 or 4 mM LiPF_6_. After 3 h of incubation, 40 OD cells were collected and lysed with zymolyase followed by two-step centrifugation to obtain crude preparations of mitochondria. Using a firefly luciferin-luciferase assay, the ATP synthetic activity of the mitochondria was determined. The relative ATP synthetic activity was normalized by each protein concentration of different samples determined using the Bradford method [44].

## 3. Results

### 3.1. Growth in LiPF_6_-, LiCl-, and NaPF_6_-Supplemented Medium

**Figure 1:**
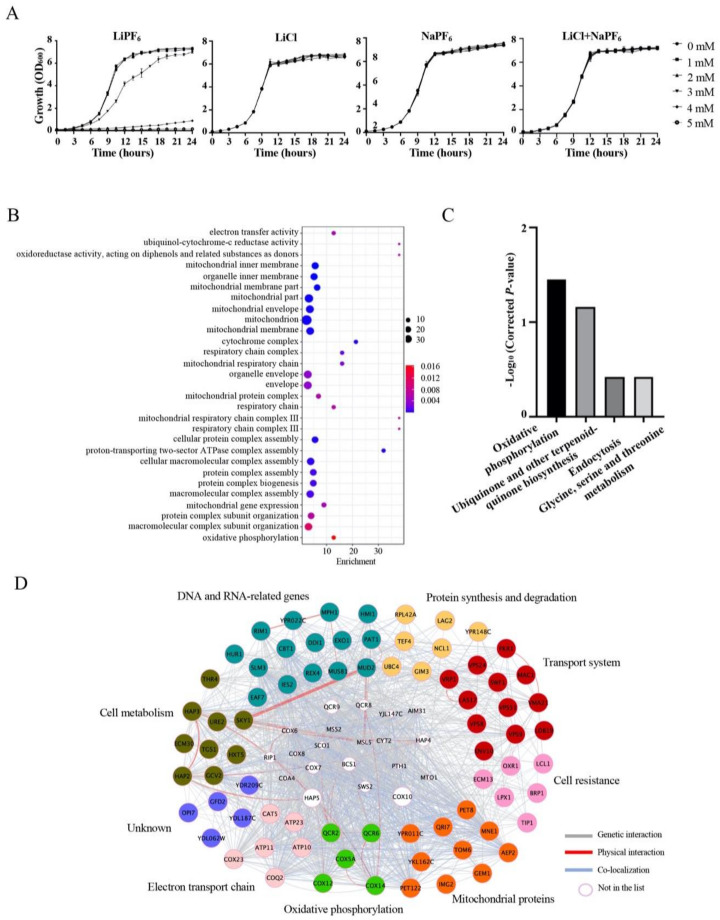
Identification of LiPF_6_-sensitive mutants by genome-wide screening. (**A**) Sensitivity of *S. cerevisiae* to LiPF_6_, LiCl, NaPF_6_, and both LiCl and NaPF_6_. Cell growth of BY4741 treated with different concentrations of LiPF_6_, LiCl, and NaPF_6_, respectively, was measured by reading absorbance at 600 nm (OD_600_) at the indicated time points. Growth curves were performed in triplicate. Growth was represented by mean OD_600_ values and error bars indicate SE. (**B**) GO term analysis for the 75 genes in Table 1, the deletion of which resulted in LiPF_6_ sensitivity. (**C**) KEGG analysis for the 75 genes in Table 1. The *p*-value was corrected using the Benjamini and Hochberg (1995) correction method [45]. (**D**) Genetic interactions, physical interactions, and co-localization of the 75 LiPF_6_-sensitive genes. Grey, red, and blue edges indicate genetic interactions, physical interactions, and co-localization, respectively. The node colors indicate different functions. SE, Standard Error; GO, Gene Ontology; KEGG, Kyoto Encyclopedia of Genes and Genomes.

We monitored cell growth of BY4741 in YPD containing different concentrations of LiPF_6_, LiCl, and NaPF_6_, ranging from 0 mM to 5 mM. Compared with the non-LiPF_6_-treated culture, the cell growth in the LiPF_6_-treated cultures did not change significantly when the LiPF_6_ concentrations were 2 mM or lower. However, cell growth was affected in cultures with LiPF_6_ concentrations of 3 mM, with a notably prolonged log phase growth (Figure 1A). Still, cells were able to grow to normal levels, similar to the controls, at 24 h. This indicated that tolerance mechanisms utilized by the cells to protect themselves from the toxicity of LiPF_6_ had been activated. In stark contrast to the growth in these moderate- and low-concentration conditions, upon exposure to higher concentrations (4 mM) cell growth was seriously inhibited, and the lag phase was substantially prolonged. Finally, when the concentration was increased to 5 mM, cell growth was completely inhibited (Figure 1A). This indicates that LiPF_6_ is very toxic to yeast cells in concentrations above 4 mM. In order to compare the toxicity of LiPF_6_ and separate the overall impact of LiPF_6_ into that from the single factors of Li^+^- or PF_6_^−^-induced toxicity, we also measured the growth of BY4741 in medium containing the same concentration of LiCl or NaPF_6_, so that each of the three solutions had the same amount of Li^+^ or PF_6_^-^ (Figure 1A). However, the sensitivity of the wild-type *S. cerevisiae* varied between the solutions in an unexpected way. BY4741 showed a much lower sensitivity to both LiCl and NaPF_6_; cell growth was not significantly affected in the presence of either LiCl or NaPF_6_, even at the maximum concentration of 5 mM (Figure 1A). In order to further verify this result, the inhibition zone of these three compounds against *S. cerevisiae* was investigated. LiPF_6_ has an obvious inhibition zone when the concentrations were 30 mM or higher, but LiCl and NaPF_6_ did not affect yeast growth at the same concentrations (Figure S1). The growth of BY4741 in liquid medium with both LiCl and NaPF_6_ added at the same time was also detected. There is no obvious growth inhibition even the concentrations of LiCl and NaPF_6_ increased to 5 mM (Figure 1A). This indicates that the LiPF_6_ electrolytes used in lithium-ion batteries have higher toxicity than either Li^+^ or PF_6_^−^ alone, at the same ionic concentration; thus, LiPF_6_ has its own unique toxicity, which is not a simple additive effect of two compounds.

### 3.2. A Genome-Wide Screen Identifies Deletion Strains with Increased Sensitivity to LiPF_6_

In order to obtain a global view of the genes involved in LiPF_6_ toxicity and tolerance in *S. cerevisiae*, a genome-wide screen of the SGA yeast deletion collection was performed. The first step was to determine the LC_50_ of LiPF_6_ on solid medium, a concentration high enough to inhibit growth but below the concentration for 100% lethality. We observed cell growth on randomly selected 96-well plates in solid medium containing 0 mM, 2 mM, 3 mM, and 4 mM LiPF_6_. In the presence of 3 mM LiPF_6_, control strains at the outer ring of the plates showed about 50% inhibition while some deletion mutants (Figure S2, red circles) exhibited notable growth inhibition, implying that these mutants were sensitive to LiPF_6_. Since the cell growth of the control strains was reduced by nearly half under this concentration, approximating the LC_50_, we screened the deletion collection for growth changes on solid plates containing 3 mM LiPF_6_. After three independent replicates, we identified 75 strains with enhanced sensitivity to LiPF_6_ (Table 1).

GO analysis of sensitive deletion mutants was performed to identify the significantly overrepresented categories of genes among the sensitive strains. In the GO result, most of the enriched genes in the deletion mutants that showed sensitivity to LiPF_6_ were associated with mitochondria or mitochondria-related function (Figure 1B). Among these, the main enriched GO terms were ubiquinol-cytochrome-c reductase activity and oxidoreductase activity, acting on the diphenols and related substances as donors, mitochondrial respiratory chain complex III, and respiratory chain complex III (Figure 1B). Through localization analysis, we noted that mitochondria-localized proteins were highly represented in our sensitive strains list, accounting for about 25% of the sensitivity-associated genes (Table S2). This indicated that mitochondria-localized proteins were the main group that responded to LiPF_6_. KEGG analysis of the sensitive strains was also performed, and four enriched pathways were found: oxidative phosphorylation; ubiquinone and other terpenoid-quinone biosynthesis; endocytosis; and glycine, serine, and threonine metabolism (Figure 1C). However, only oxidative phosphorylation was enriched with a cut-off *p*-value of <0.05. Oxidative phosphorylation was the functional category for four genes: COX5A, COX12, QCR2, and QCR6. All of these genes participate in mitochondrial respiration and ATP synthesis [46]. In summary, the sensitive mutants had defects in mitochondrial function, suggesting an important role for mitochondria in LiPF_6_-induced toxicity.

In addition, functional classification of the genes that, upon deletion, resulted in higher sensitivity to LiPF_6_ was performed according to the functional description from the Saccharomyces Genome Database. The 75 genes, with the exception of five uncharacterized genes, were classified into eight groups: oxidative phosphorylation, electron transport chain, mitochondrial proteins, cell resistance, transport system, protein synthesis and degradation, DNA and RNA-related genes, and cell metabolism (Figure 1D and Table 1). Genetic and physical interaction networks were also analyzed, which provided information on the functional association between the genes of interest. This analysis demonstrated that over 22% (17/75) of the encoded gene products are ones that are reported to have physical interactions with each other (Figure 1D, red lines). There were also many genetic interactions between the 75 genes that were not part of the physical interaction networks (Figure 1D, grey lines). Co-localization analysis revealed that most of the encoded gene products were co-localized with other proteins (Figure 1D, blue lines). This indicated that many of these genes were not only associated in terms of functional processes, but were also physically bound to each other, to cooperatively participate in the modulation of LiPF_6_ toxicity.

### 3.3. Oxidative Phosphorylation-Related Genes Are Required for Tolerance to LiPF_6_

**Figure 2:**
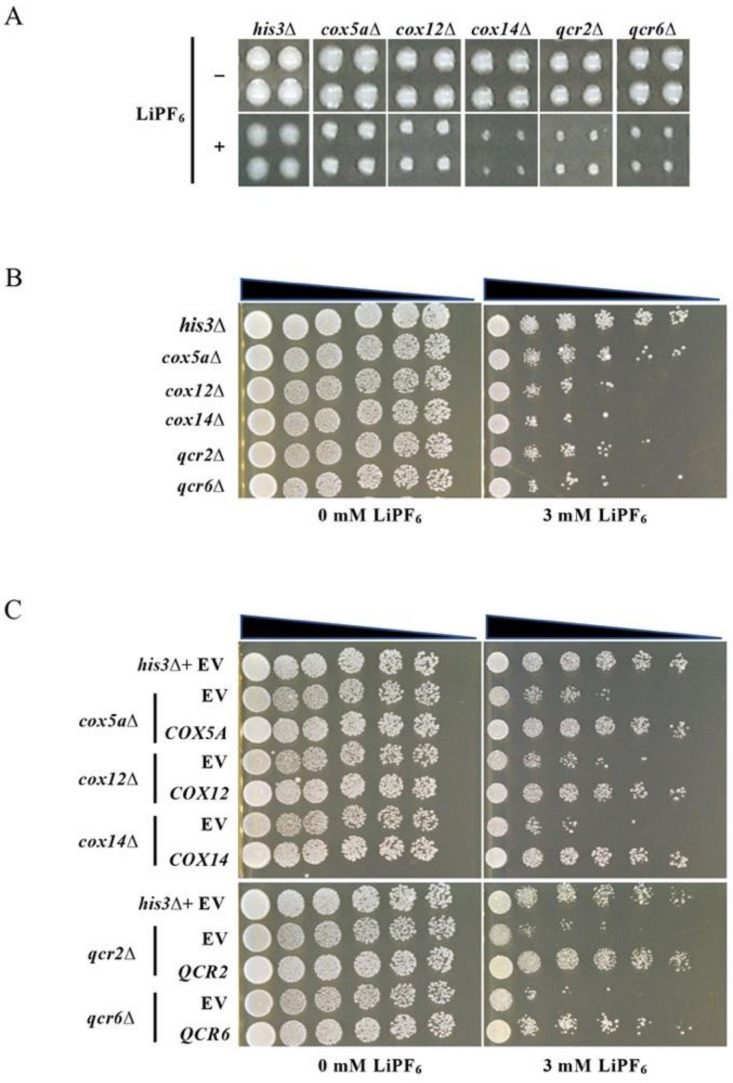
Oxidative phosphorylation-related gene deletion strains show increased sensitivity to LiPF_6_. (**A**) Phenotypes of the SGA control strain (*MATa his3Δ::kanMX4*) and deletion mutants in our screen. Each strain was arranged in quadruplicate. (**B**) Spot test to verify the screening results. The control strain and *cox5a∆*, *cox12∆*, *cox14∆*, *qcr2∆*, and *qcr6∆* strains were grown to mid-log phase in YPD medium and then diluted to an OD_600_ of 0.5. Cells were serially diluted onto YPD agar plates either containing 3 mM LiPF_6_ or no LiPF_6_. Plates were photographed after 48 h of incubation at 30 °C. Images shown are representative of triplicates. (**C**) Spot test of the complementation strains. Deletion mutant strains transformed with empty vector or plasmid expressing the corresponding genes were grown to mid-log phase in YPD medium before diluting to an OD_600_ of 0.5 and cells were serially diluted onto YPD plates with 3 mM LiPF_6_ or without LiPF_6_. The control strain transformed with empty vector served as a control. Images were representative of triplicates. SGA, Synthetic Genetic Array; YPD, Yeast Peptone Dextrose.

GO and KEGG analysis indicated that mitochondria were the main organelles involved in the response to LiPF_6_, so we focused on the oxidative phosphorylation pathway, which occurs in the mitochondria. Of the 75 genes from the sensitive mutant strains identified during the screening, four were the components of the oxidative phosphorylation pathway. The deletion mutants of these genes were not, however, responsive to LiCl, even at a high concentration (0.4 M) [29], implying that the oxidative phosphorylation pathway might be specifically involved in cell tolerance to LiPF_6_. Ubiquinol-cytochrome-c reductase subunit 2 (Qcr2) and subunit 6 (Qcr6) are subunits of the electron transfer chain complexes III, namely ubiquinol cytochrome-c reductase complex [47,48]. Cox5a and Cox12 are the subunits of cytochrome c oxidase (COX), which is part of the complex IV of the mitochondrial electron transport chain [49]. In addition, we also tested another gene, cytochrome c oxidase assembly factor 14 (COX14). Although it is not included in oxidative phosphorylation pathway in KEGG analysis, it is associated with cytochrome c oxidase assembly [50,51] and we tested these genes together. In our screen, deletion of any of these genes conferred cell sensitivity to LiPF_6_ (Figure 2A). In order to clarify whether these specific genes indeed modulate LiPF_6_ toxicity, spot test assays for each of the mutants were carried out to validate the results of the large-scale screen. As shown in Figure 2B, *cox5a∆*, *cox12∆*, *cox14∆*, *qcr2∆*, and *qcr6∆* mutants displayed significant compromise in growth on plates with LiPF_6_. Plasmids from the MoBY ORF 1.0 Library expressing each of these genes were then transformed into their corresponding deletion mutants to confirm whether the LiPF_6_-sensitive phenotype is due to the specific disruption of these genes. The mutants *cox5a∆*, *cox12∆*, *cox14∆*, *qcr2∆*, and *qcr6∆* carrying the complementary plasmid exhibited normal growth, similar to that of the control strain carrying an empty vector, whereas the deletion strains carrying empty vectors continued to exhibit sensitivity to LiPF_6_ (Figure 2C). These results suggested that the sensitive phenotype of the mutants was indeed caused by deletion of the genes encoding Cox5a, Cox12, Cox14, Qcr2, and Qcr6. As Cox5a, Cox12, Cox14, Qcr2, and Qcr6 are critical components of the oxidative phosphorylation pathway and are essential for mitochondrial energy production to support most of the metabolic activities in cells, it is speculated that normal mitochondrial function maintenance is required for cellular tolerance to LiPF_6_.

### 3.4. High Concentration of LiPF_6_ Alters Mitochondrial Morphology, Induces ROS Accumulation, and Reduces ATP Levels

**Figure 3:**
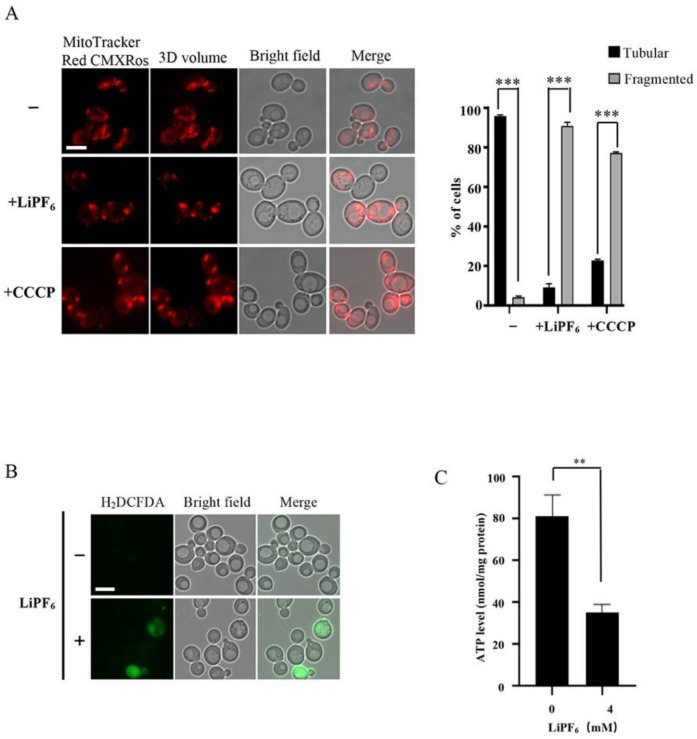
Effects of LiPF_6_ on mitochondrial morphology, ROS, and ATP synthesis. (**A**) Mitochondrial morphology of BY4741 was observed with or without LiPF_6_. A total of 10 μM CCCP-treated cells served as the positive control. Mitochondria stained with MitoTracker Red CMXRos are shown on the left; 3D volume images of mitochondria are shown in the second column; bright-field micrographs are shown in the third column; and merged images are shown on the right. “−”: without LiPF_6_ and CCCP; “+ LiPF_6_”: with 4 mM LiPF_6_; “+ CCCP”: with 10 μM CCCP. Scale bar represents 5 μm. The percentage of cells exhibiting tubular or fragmented mitochondria was calculated. At least 200 cells of each sample were used for quantitation. Error values indicate the SE from three independent experiments. ***, *p* < 0.001. (**B**) Intracellular ROS were detected in BY4741 in the absence or presence of LiPF_6_. ROS signal stained with H_2_DCFDA was shown on the left; bright-field micrographs are shown in the middle; and merged images are shown on the right. “−”: without LiPF_6_; “+”: with 4 mM LiPF_6_. Scale bar represents 5 μm. (**C**) Mitochondrial ATP synthesis was measured after incubation with 0 or 4 mM LiPF_6_. The vertical axis represents the ATP content per mg protein. Error bars indicate the SE from three independent experiments. **, *p* < 0.01, Student’s *t*-test. CCCP, Carbonyl cyanide m-chlorophenylhydrazone; ROS, Reactive Oxygen Species; SE, Standard Error.

Mitochondria are essential organelles with multiple functions in eukaryotic cells. They are responsible for the generation of ATP, which serves as an energy source for numerous critical cellular activities, and are involved in apoptosis, ion homeostasis, and signal transduction [52,53,54]. During the process of oxidative phosphorylation, mitochondria are also associated with the generation and management of ROS, which was the main source of intracellular ROS [55,56]. In our screening, among the LiPF_6_-sensitive strains, it was mitochondrial mutants that were most enriched, indicating that mitochondria may play essential roles in modulating LiPF_6_-associated toxicity. The yeast growth in YPG medium in the absence or presence of LiPF_6_ was measured, in which glycerol serves as the single carbon source and cells depend on mitochondria to generate energy. Compared to the growth in YPD with 4 mM LiPF_6_, which would resume after 60 h, the yeast growth in YPG in the presence of 4 mM LiPF_6_ was completely inhibited (Figure S3). Since utilization of the nonfermentable carbon source glycerol requires mitochondrial function, it provided evidence for the toxic effects of LiPF_6_ on mitochondria. Therefore, we then analyzed mitochondrial morphology changes in response to LiPF_6_ treatment. Cells treated with carbonyl cyanide m-chlorophenylhydrazone (CCCP), a mitochondrial uncoupler, served as the positive control (Figure 3A). Under normal conditions, cells displayed a dynamic branched tubular mitochondrial network, as expected from previous studies [57]. In the presence of CCCP or 4 mM LiPF_6_, the tubular mitochondria were disrupted into fragmented shapes (Figure 3A). We quantified the fragmented mitochondria before and after LiPF_6_ incubation and found that ~90% of cells displayed fragmented mitochondria after LiPF_6_ exposure (Figure 3A), even higher than that of positive control. In order to further verify the effect of LiPF_6_ on mitochondrial morphology, we used a mitochondrial matrix protein Ilv3 fused to GFP as a mitochondria marker to observe the morphological alterations [57]. Similarly, under normal conditions, a tubular mitochondrial network was observed. However, after LiPF_6_ treatment, about 95% of the cells displayed a fragmented GFP signal (Figure S4). Thus, the mitochondria were drastically reshaped when subjected to high concentrations of LiPF_6_.

As damaged mitochondria are the main source of intracellular ROS [58], we sought to determine whether LiPF_6_ could induce ROS in yeast. Mid-log phase cells were treated with 1 mM LiPF_6_ in synthetic complete (SC) liquid medium, and H_2_DCFDA was utilized in the measurement of ROS levels [59]. As shown in Figure 3B, almost no ROS signal was detected in the untreated cells, while in the presence of LiPF_6_, the wild-type strain exhibited a positive ROS signal throughout the cell, showing that LiPF_6_ can induce oxidative stress (Figure 3B). Furthermore, we tested whether the production of ATP was affected by LiPF_6_. Crude mitochondrial fraction was isolated from wild-type strains treated with 0 mM or 4 mM LiPF_6_, and then ATP synthesis was measured. As shown in Figure 3C, the ATP synthesis ability of mitochondria isolated from LiPF_6_-treated cultures was significantly impaired as the ATP levels decreased compared with that of the control.

### 3.5. Oxidative Phosphorylation-Related Genes Are Required for Counteraction of LiPF_6_-Induced ROS

**Figure 4:**
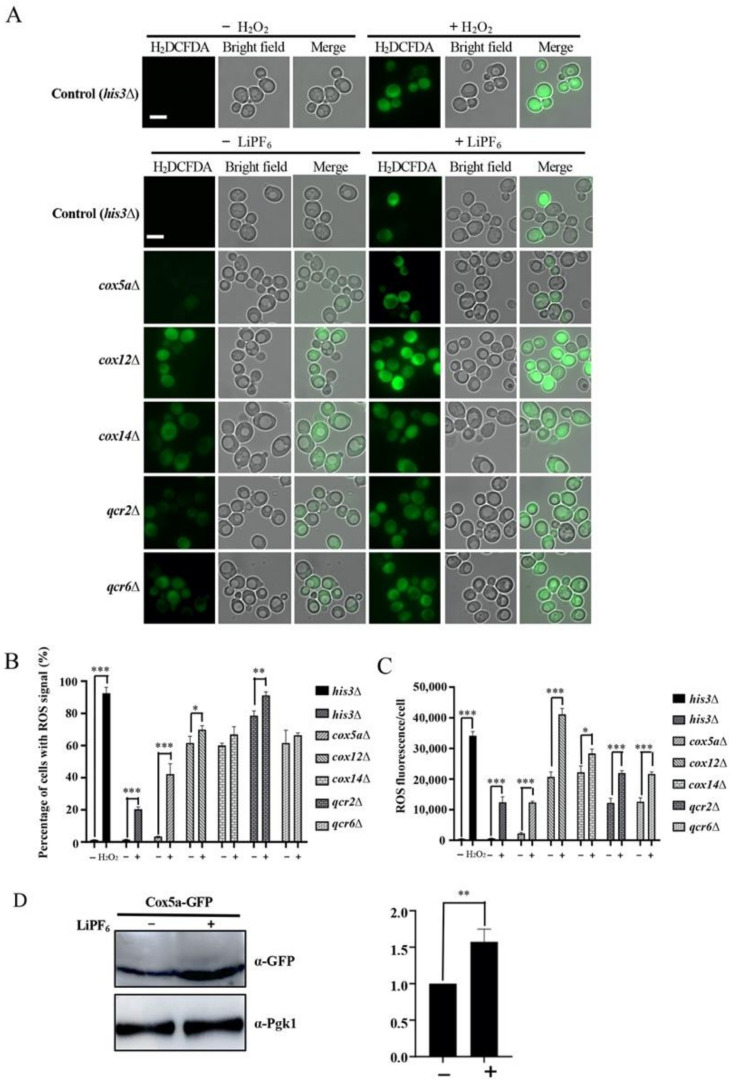
Oxidative phosphorylation-related genes are required for the regulation of LiPF_6_-induced ROS. (**A**) The presence of ROS in BY4741 and the deletion mutant strains in medium with or without LiPF_6_ was determined. The H_2_O_2_-treated SGA control strain (*his3Δ*) served as a positive control. Left, ROS signal; middle, bright-field micrographs; right, merged images. Scale bar represents 5 μm. (**B**) Quantifications of H_2_DCFDA-positive cells. Three independent biological experiments were carried out, and for each replicate, a minimum of 200 cells were counted. The vertical axis represents the percentage of cells with a ROS signal, and the horizontal axis represents the different strains. Error bars indicate SE. ***, *p* < 0.001, **, *p* < 0.01, *, *p* < 0.05, Student’s *t*-test. (**C**) Quantifications of fluorescence intensity per cell. Three independent biological experiments were carried out and for each replicate, and a minimum of 200 cells were counted. The vertical axis represents the fluorescence intensity per cell, and the horizontal axis represents the different strains. Error bars indicate SE. ***, *p* < 0.001, *, *p* < 0.05, Student’s *t*-test. (**D**) Western bolt analysis of Cox5a before and after LiPF_6_ treatment. Pgk1 served as a loading control. Accumulation levels of Cox5a protein were quantified using ImageJ software. Error bars indicate SE. **, *p* < 0.01, Student’s *t*-test. ROS, Reactive Oxygen Species; SE, Standard Error.

In order to explain why deletion of oxidative phosphorylation-related genes leads to cells to exhibit sensitivity to LiPF_6_, we tested the ROS levels in deletion strains when treated with LiPF_6_. The H_2_O_2_-treated SGA control strain (*his3Δ*) was used as a positive control. An untreated control strain (*his3Δ*) was used as a negative control. As expected, after treated with H_2_O_2_, strong fluorescent signals were accumulated in the cells (Figure 4A). Like the control strain, a limited ROS signal was detected in mutant *cox5a∆* in the absence of LiPF_6_. However, upon exposure to LiPF_6_, *cox5a∆* accumulated substantial ROS (Figure 4A). The percentage of ROS indicator-stained cells was counted, and is shown in Figure 4B. For the control strain exposed to LiPF_6_, about 20% of the cells accumulated ROS. However, for the *cox5a∆* strain, the proportion increased to approximate 40%. Thus, Cox5a was required to partially counteract the LiPF_6_-induced ROS. In the other four deletion mutants (*cox12∆*, *cox14∆*, *qcr2∆*, and *qcr6∆*), deletion of these genes caused production of ROS in more than 50% of the cells under normal conditions (Figure 4A,B), suggesting that these four genes were critical for intracellular ROS balance under normal physiological conditions. Upon exposure to LiPF_6_, although the proportion of *cox12∆*, *cox14∆*, *qcr2∆*, and *qcr6∆* cells with ROS signal was not increased or slightly increased compared with untreated cells (Figure 4B), the average fluorescence intensity per cell in each mutant was remarkably increased, and was much higher than that of the control strain (Figure 4C). This suggested that Cox12, Cox14, Qcr2, and Qcr6 are also involved in the mediation of LiPF_6_-induced ROS.

Then, the protein expression levels of these genes were detected before and after LiPF_6_ treatment. Cox5a protein levels were remarkably increased after LiPF_6_ treatment (Figure 4D). Increased Cox5a might help cells to maintain ROS at a low level. The levels of the other four proteins were not significantly changed or cannot be detected (Figure S5). In summary, deletion of the oxidative phosphorylation-related genes caused a high level of ROS accumulation in cells treated with LiPF_6_. This may be one of the reasons why deletion of these genes increased sensitivity to LiPF_6_.

### 3.6. Deletion of Oxidative Phosphorylation-Related Genes Alters ATP Synthesis Abilities under High Concentration of LiPF_6_ Treatment

**Figure 5:**
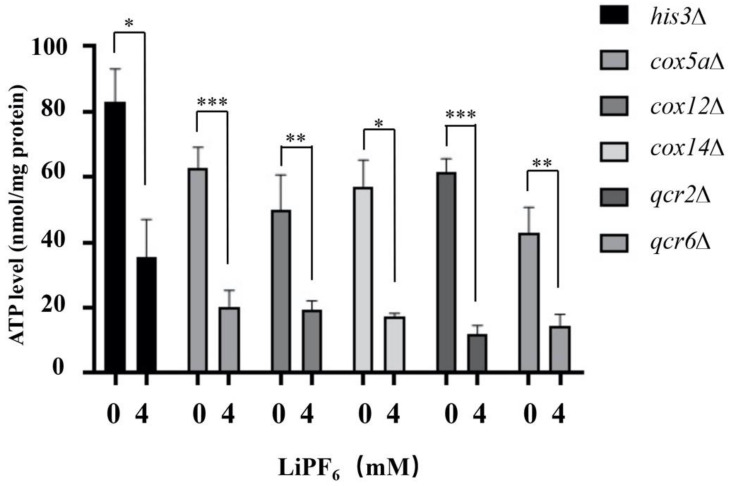
Deletion of the oxidative phosphorylation-related genes aggravated the decrease in ATP production under LiPF_6_ treatment. ATP synthesis abilities were compared between the control strain (*his3Δ*) and the deletion mutants. The vertical axis represents the ATP content per mg protein. The data shown represent averages of three experiments, and error bars indicate SE. ***, *p* < 0.001, **, *p* < 0.01, *, *p* < 0.05, Student’s *t*-test. YPD, Yeast Peptone Dextrose; SE, Standard Error.

To examine whether deletion of these five oxidative phosphorylation-related genes could affect mitochondrial ATP synthetic activity in the presence of LiPF_6_, ATP synthesis was compared between the control strain (*his3Δ*) and the deletion strains. Under normal physiological conditions, the ATP synthesis abilities of the deletion mutants were lower than that in the control strain (Figure 5). When exposed to 4 mM LiPF_6_, the control strain showed about a 45% decrease in ATP production. However, for the deletion strains, the gap between the treated and untreated cells was widened (Figure 5). Furthermore, the ATP synthesis in these five deletion strains in the presence of LiPF_6_ was less than 25 nmol/mg, which was much lower than the 35 nmol/mg in the control strain (Figure 5). Thus, we can see that Cox5a, Cox12, Cox14, Qcr2, and Qcr6 were indispensable for maintaining the ATP levels when exposed to LiPF_6_. Higher ATP levels are beneficial in helping cells survive when they are exposed to LiPF_6_.

Among the sensitive strains, there were three other genes involved in mitochondrial ATP synthesis, ATP10, ATP11, and ATP23, which belong to the mitochondrial F1F0 ATP synthase. We speculate that these genes might also be important for the tolerance to LiPF_6_ because they are critical for cellular ATP content maintenance. Spot test assays of these mutants and complementation strains were carried out to confirm the result of high-throughput screening. Data in Figure S6A,B indicated that deletion of these three genes result in sensitivity to LiPF_6_. A further ATP assay suggested that deletion of these mitochondrial F1F0 ATP synthase-related genes results in decreased ATP levels in the cells compared with the control strain under normal conditions. For *atp10Δ* and *atp23Δ*, in the presence of LiPF_6_, the ATP content is further decreased and lower than that of control strain (Figure S6C). This could be the potential reason for their LiPF_6_ sensitivity.

### 3.7. Oxidative Phosphorylation-Related Mutants Were Specifically Hypersensitive to LiPF_6_

**Figure 6:**
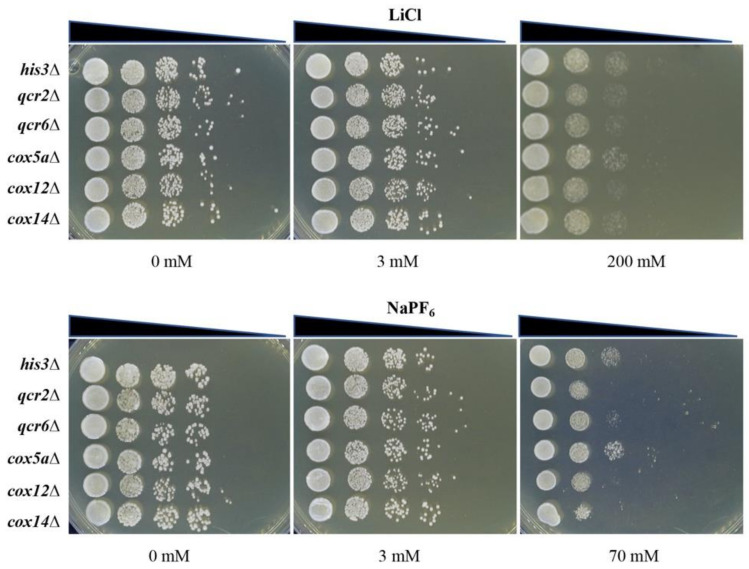
Oxidative phosphorylation-related genes are not hypersensitive to LiCl or NaPF_6_. The SGA control strain and deletion strains were grown to mid-log phase in YPD medium before diluting to an OD_600_ of 0.5. Cultures were serially diluted onto YPD plates containing different concentrations of LiCl or NaPF_6_. Plates were incubated at 30 °C and photographed. SGA, Synthetic Genetic Array; YPD, Yeast Peptone Dextrose.

In order to test whether the identified oxidative phosphorylation-related genes (COX5A, COX12, COX14, QCR2, and QCR6) were specifically responsive to LiPF_6_, spot test assays were performed to examine deletion mutant growth on the LiCl and NaPF_6_ plates. First, we observed the cell growth on 3 mM LiCl- and NaPF_6_-containing plates, which have the same number of moles of Li^+^ and PF_6_^−^ as 3 mM LiPF_6_. The data demonstrated no significant difference between the control and deletion strains when exposed to Li^+^ or PF_6_^−^ ions in the form of LiCl and NaPF_6_ (Figure 6). When the concentration of NaPF_6_ was increased to 70 mM, the *qcr2∆*, *qcr6∆*, *cox12∆*, and *cox14∆* mutants exhibited compromised growth compared with the control strain. However, the *cox5a∆* mutant was still not suppressed. Likewise, the growth of the *qcr2∆*, *qcr6∆*, *cox5a∆*, *cox12∆*, and *cox14∆* mutants on LiCl plates showed no difference compared with the control strain despite the concentration increasing to 200 mM (Figure 6), which was consistent with previous published results [29]. These results indicated that Cox5a specifically responds to LiPF_6_, and that, although the other four gene mutants were cross-sensitive to NaPF_6_, a high concentration of NaPF_6_ was required to elicit observable effects.

## 4. Discussion

LiPF_6_ is one of the leading electrolytes in lithium-ion batteries and is toxic to the environment and organisms [4]. It is important to reveal the mechanism underlying the toxicity of LiPF_6_ and to decipher the specific response of cells to it. Our results revealed that the yeast was more sensitive to LiPF_6_ than to LiCl or NaPF_6_. Physiological and morphological analysis revealed that mitochondrial damage, oxidative stress, and ATP imbalance were the driving factors governing LiPF_6_-induced toxicity. A genome-wide screening of the yeast deletion collection identified 75 mutants that showed sensitivity to LiPF_6_. Among these, the oxidative phosphorylation pathway was the most enriched pathway, per the KEGG database, and genes in this pathway were specifically hypersensitive to LiPF_6_. In the presence of LiPF_6_, mutants with deletions of these genes exhibited higher ROS production and reduced ATP production compared with the control strain, and this might explain their sensitivity to LiPF_6_. Our study not only identified the process by which LiPF_6_ induces cytotoxicity, but also elucidated the specific genes that confer cell tolerance to LiPF_6_.

Yeast has a high tolerance to Li^+^, as shown by a previous study, in which researchers used 0.1 M LiCl to identify its hypersensitive mutants [29]. Likewise, in our study, we also found that a low concentration of NaPF_6_ (5 mM) did not affect yeast growth. In contrast, 5 mM LiPF_6_ completely inhibited yeast growth. This indicates that LiPF_6_ was more toxic than LiCl or NaPF_6_ in yeast, and that the cytotoxicity induced by LiPF_6_ might be different from that induced by LiCl or NaPF_6_. Zhao et al. identified 114 LiCl-sensitive mutants in a genome-scale genetic screening. A large number of the mutant genes were identified as being involved in sporulation and meiosis, and vacuolar protein sorting; these are the two major cellular processes affected by LiCl [29]. These genes were found to be associated with intracellular lithium content and ion homeostasis, which might be involved in sensitivity to lithium stress [29]. In our study, mitochondrial genes and genes related to mitochondrial processes were the genes found to be most enriched, and represented the major cellular responses to LiPF_6_. We compared the 75 LiPF_6_-sensitive genes identified here (Table 1) with the 114 lithium-sensitive mutants reported previously. We observed that four LiPF_6_-sensitive genes were also involved in sensitivity to LiCl. Three of these encode proteins involved in vacuolar protein sorting (Vps8, Vps9, and Vps51). Vps8 functions in protein targeting during late endosome-to-vacuole transport [60,61]. Vps9 is involved in Golgi-endosome trafficking and sorting through the multivesicular body [62,63]. Vps51 is required for the recycling of proteins from endosomes to the late Golgi [64,65]. Deletion of Vps8, Vps9, and Vps51 leads to accumulation of intracellular lithium contents, implying that these genes are critical for ion homeostasis [29]. Regarding Vps8 and Vps9, mutants *vps8∆* and *vps9∆* are sensitive to LiCl only when concentrations are 0.4 M or higher. In contrast, the phenotypes of mutants *vps8∆* and *vps9∆* included sensitivity to 3 mM LiPF_6_ (a concentration that should not induce lithium stress in yeast). This suggests that the sensitivity to LiPF_6_ might not be attributable to lithium homeostasis disruption. Thus, although some strains with deletions of vacuolar protein sorting genes exhibited cross-sensitivity to LiCl, this might be due to alteration of other biological processes, causing the yeast cells to become sensitive to LiPF_6_.

Fluorescence microscope observations indicated that LiPF_6_ induced fragmentation of mitochondria and accumulation of ROS, which could result in lipid, protein, and DNA peroxidation [66,67,68]. ATP synthetic activity measurement using the isolated crude mitochondrial fraction indicated that LiPF_6_ impairs ATP synthesis. Moreover, LiPF_6_ treatment causes more toxicity to yeast cells when cultured in respiratory medium. It also provides evidence for the negative effect of LiPF_6_ on mitochondria. Previous studies reported that high concentrations of LiCl and Li_2_CO_3_ impaired mitochondria complex II and IV activity, enhanced ROS formation, lowered mitochondrial membrane potential, and induced cytochrome c release from the mitochondria to the cytosol [14,69,70,71]. In addition, a recent review also summarized research indicating that an overdose of fluoride can induce mitochondrial damage, affect the regulation of intracellular redox homeostasis, and activate endoplasmic reticulum stress and apoptosis [72]. Thus, LiPF_6_ might have a similar mode of action, via effects on mitochondria, to those of lithium chloride, lithium carbonate, or fluoride. However, as the molar quantity of Li^+^ and PF_6_^−^ of LiPF_6_ in our study was much lower than that in the above-discussed studies, the effect of LiPF_6_ on mitochondria was much stronger.

The mitochondrial respiratory chain consists of the NADH dehydrogenase complex, succinate dehydrogenase complex, cytochrome c reductase complex, and cytochrome c oxidase complex, as well as the ATP synthase complex, together with ubiquinone and cytochrome c, which act as electron carriers [73]. The mitochondrial electron transport chain plays a major role in ATP production, but this process is accompanied by the generation of ROS [74]. In our genome-wide screening, the cytochrome c reductase complex mutants *qcr2∆* and *qcr6∆*, the cytochrome c oxidase complex mutants *cox5a∆*, *cox12∆*, and *cox14∆*, the ATP synthase complex mutant *atp10∆*, *atp11∆*, and *atp23∆* were identified to be sensitive to LiPF_6_. Previous studies have reported that deletion of these genes results in different degrees of mitochondrial dysfunction [55]. However, *S. cerevisiae* does not strictly depend on the function of mitochondria when cultured in YPD medium. Under normal physiological conditions, *cox5a∆*, *cox12∆*, *cox14∆*, *qcr2∆*, *qcr6∆*, *atp10∆*, *atp11∆*, and *atp23∆* had similar growth to or weaker growth than that of the control strain, as well as in the ATP synthesis ability. In the presence of LiPF_6_, however, cell growth and ATP synthesis were remarkably reduced in deletion mutants. This gave us a clue that mitochondrial efficiency and sufficient ATP content are critical for tolerance to LiPF_6_. ROS measurement also indicated that COX5A deletion leads to an increased proportion of cells with ROS signal compared to the control strain. For the other four oxidative phosphorylation-related genes, although deletion mutants of these genes did not significantly affect the proportion of cells with a ROS signal after LiPF_6_ treatment, the average fluorescence intensity per cell was increased. From these data, we speculated that the oxidative phosphorylation-related genes were critical for the counteraction of LiPF_6_-induced ROS accumulation and ATP reduction, processes that might be beneficial for cell survival after exposure to LiPF_6_. More importantly, western blot analysis suggested that the Cox5a protein was accumulated after LiPF_6_ exposure. Combined with the results from the spot test of LiCl and NaPF_6_, we concluded that *cox5a∆* was specifically sensitive to LiPF_6_ and the increased protein expression of Cox5a is a mechanism by which yeast cells can overcome the toxicity of LiPF_6_.

Taken together, our study revealed the cytotoxicity of the lithium-ion battery electrolytes LiPF_6_, and identified the genes related to oxidative phosphorylation as critical for conferring resistance to LiPF_6_. This work will provide valuable information about the toxicity mechanisms of industrial products and will give researchers valuable information to be used in policy choices in the relevant industry fields.

## Figures and Tables

**Table 1:** The 75 genes deleted in the LiPF_6_-sensitive mutants, as identified from the genome-wide screen.

ORF	Gene	Score 1 ^a^	*p*-Value 1 ^a^	Score 2 ^a^	*p*-Value 2 ^a^	Score 3 ^a^	*p*-Value 3 ^a^	Location ^b^
**Oxidative Phosphorylation**								
YNL052W	COX5A	−0.3751	0.00006	−0.2880	0.00027	−0.3322	0.00011	mitochondrion
YLR038C	COX12	−0.3392	0.00002	−0.7994	0.00339	−0.7440	0.01456	cytoplasm
YML129C	COX14	−0.5864	0.00055	−0.7346	0.00388	−0.3517	0.00018	mitochondrion
YPR191W	QCR2	−0.5095	0.00365	−0.7160	0.00026	−0.3503	0.00001	mitochondrion
YFR033C	QCR6	−0.2124	0.00002	−0.5472	0.00001	−0.2844	0.00006	cytoplasm
**Electron Transport Chain**								
YNL315C	ATP11	−0.4236	0.00001	−0.4884	0.00003	−0.2834	0.00001	mitochondrion
YNR020C	ATP23	−0.4009	0.00000	−0.4283	0.00013	−0.3016	0.00001	-
YLR393W	ATP10	−0.3061	0.00000	−0.3744	0.00107	−0.4161	0.00003	mitochondrion
YOR125C	CAT5	−0.6673	0.00062	−0.6531	0.00018	−0.4810	0.00038	-
YNR041C	COQ2	−0.5151	0.00196	−0.6484	0.00043	−0.4307	0.00005	mitochondrion
YHR116W	COX23	−0.4963	0.00000	−0.6415	0.00326	−0.4725	0.00035	cytoplasm
**Mitochondrial Proteins**								
YMR282C	AEP2	−0.6256	0.00030	−0.6545	0.00012	−0.4035	0.00046	mitochondrion
YNL003C	PET8	−0.6297	0.00277	−0.6204	0.01065	−0.4815	0.00005	mitochondrion
YAL048C	GEM1	−0.5227	0.00222	−0.4628	0.00008	−0.3097	0.00008	-
YPR011C	-	−0.3696	0.00006	−0.3274	0.00013	−0.2720	0.00212	mitochondrion
YOR045W	TOM6	−0.2975	0.00012	−0.2318	0.00004	−0.3106	0.00001	mitochondrion
YKL162C	-	−0.4414	0.00001	−0.3662	0.00005	−0.2634	0.00001	mitochondrion
YOR350C	MNE1	−0.2384	0.00003	−0.6479	0.00014	−0.2888	0.00001	mitochondrion
YDL104C	QRI7	−0.6281	0.00128	−0.6104	0.00025	−0.3629	0.00001	mitochondrion
YER153C	PET122	−0.2664	0.00032	−0.6257	0.00064	−0.3802	0.00001	mitochondrion
YCR071C	IMG2	−0.5618	0.00349	−0.5690	0.00003	−0.3813	0.00003	mitochondrion
**DNA and RNA-Related Genes**								
YCR028C-A	RIM1	−0.5714	0.00325	−0.5815	0.00024	−0.2990	0.00003	mitochondrion
YKL208W	CBT1	−0.4650	0.00064	−0.8063	0.00017	−0.4649	0.00002	mitochondrion
YOL080C	REX4	−0.5478	0.00000	−0.5925	0.00001	−0.4610	0.00001	nucleolus, nucleus
YDL033C	SLM3	−0.4263	0.00014	−0.5773	0.00007	−0.4009	0.00002	mitochondrion
YKL074C	MUD2	−0.3940	0.00001	−0.2244	0.00011	−0.2730	0.00001	cytoplasm, nucleus
YOR033C	EXO1	−0.6815	0.00162	−0.5806	0.00015	−0.2738	0.00001	nucleus
YNL215W	IES2	−0.4173	0.00006	−0.3761	0.00000	−0.2594	0.00003	nucleus
YDR386W	MUS81	−0.2506	0.00000	−0.3685	0.00003	−0.2387	0.00001	-
YIR002C	MPH1	−0.3117	0.00000	−0.2264	0.00001	−0.5003	0.00021	cytoplasm, nucleus
YOL095C	HMI1	−0.6735	0.00339	−0.5763	0.00010	−0.2354	0.00002	-
YPR022C	-	−0.3192	0.00020	−0.3845	0.00000	−0.4943	0.00003	cytoplasm, nucleus
YNL136W	EAF7	−0.4389	0.00004	−0.2674	0.00001	−0.2094	0.00001	nucleus
YER143W	DDI1	−0.3362	0.00000	−0.2392	0.00001	−0.2629	0.00002	cytoplasm
YCR077C	PAT1	−0.3727	0.00008	−0.3601	0.00001	−0.3147	0.00005	cytoplasm
YGL168W	HUR1	−0.3131	0.00001	−0.5268	0.00000	−0.4062	0.00001	-
**Transport System**								
YAL002W	VPS8	−0.6784	0.00002	−0.6423	0.00002	−0.4657	0.00001	endosome
YML097C	VPS9	−0.6179	0.00001	−0.5143	0.00003	−0.5346	0.00008	cytoplasm
YKR020W	VPS51	−0.4877	0.00012	−0.4249	0.00002	−0.3440	0.00004	punctate composite
YKL041W	VPS24	−0.4652	0.00000	−0.4097	0.00026	−0.2860	0.00006	punctate composite, endosome
YOR322C	LDB19	−0.6997	0.00006	−0.4052	0.00000	−0.2972	0.00003	cytoplasm, late Golgi
YLR065C	ENV10	−0.2237	0.00003	−0.2707	0.00001	−0.3896	0.00009	ambiguous
YMR021C	MAC1	−0.5325	0.00027	−0.5749	0.00014	−0.5273	0.00029	cytoplasm, nucleus
YGR105W	VMA21	−0.5096	0.00006	−0.4375	0.00002	−0.3984	0.00002	vacuole
YDR126W	SWF1	−0.4926	0.00004	−0.2602	0.00000	−0.3249	0.00002	-
YMR123W	PKR1	−0.2993	0.00000	−0.2286	0.00002	−0.2083	0.00005	ER
YOR181W	LAS17	−0.2113	0.00001	−0.2026	0.00002	−0.2052	0.00002	actin
YLR337C	VRP1	−0.5067	0.00006	−0.3804	0.00000	−0.6244	0.00002	punctate composite, actin
**Cell Metabolism**								
YMR189W	GCV2	−0.2085	0.00000	−0.4420	0.00030	−0.5500	0.00002	mitochondrion
YGL237C	HAP2	−0.5162	0.00004	−0.2985	0.00000	−0.3660	0.00000	nucleus
YCR053W	THR4	−0.2961	0.00004	−0.3573	0.00001	−0.3767	0.00016	cytoplasm, nucleus
YPL157W	TGS1	−0.4255	0.00001	−0.2466	0.00001	−0.2310	0.00002	nucleolus
YMR216C	SKY1	−0.6509	0.00035	−0.5810	0.00003	−0.5751	0.00002	cytoplasm
YLR436C	ECM30	−0.2724	0.00002	−0.4312	0.00000	−0.4328	0.00002	cytoplasm
YHR096C	HXT5	−0.2206	0.00004	−0.2280	0.00002	−0.2911	0.00003	-
YBL021C	HAP3	−0.3353	0.00082	−0.2217	0.00000	−0.3064	0.00000	cytoplasm, nucleus
YNL229C	URE2	−0.2639	0.00012	−0.3145	0.00001	−0.2208	0.00000	cytoplasm
**Protein Synthesis and Degradation**								
YNL162W	RPL42A	−0.2885	0.00000	−0.2105	0.00000	−0.2294	0.00000	cytoplasm
YBL024W	NCL1	−0.5915	0.00002	−0.5668	0.00001	−0.4087	0.00007	nucleus
YPR148C	-	−0.4126	0.00003	−0.3759	0.00002	−0.4687	0.00003	punctate composite
YKL081W	TEF4	−0.3932	0.00001	−0.3216	0.00003	−0.3571	0.00000	cytoplasm
YBR082C	UBC4	−0.2783	0.00002	−0.3556	0.00001	−0.3065	0.00000	cytoplasm, nucleus
YOL025W	LAG2	−0.3460	0.00001	−0.2428	0.00000	−0.3409	0.00001	-
YNL153C	GIM3	−0.2539	0.00004	−0.2300	0.00002	−0.4280	0.00005	cytoplasm
**Cell Resistance**								
YOR084W	LPX1	−0.4131	0.00000	−0.3710	0.00029	−0.2318	0.00011	ambiguous
YPL196W	OXR1	−0.3809	0.00001	−0.2965	0.00000	−0.2073	0.00000	-
YBL043W	ECM13	−0.4461	0.00002	−0.4689	0.00000	−0.3406	0.00001	-
YPL056C	LCL1	−0.3480	0.00005	−0.3583	0.00004	−0.3946	0.00002	-
YBR067C	TIP1	−0.2071	0.00006	−0.2896	0.00003	−0.2285	0.00000	ER
YGL007W	BRP1	−0.7219	0.00031	−0.7044	0.00002	−0.6213	0.00005	-
**Unknown**								
YDL062W		−0.4626	0.00485	−0.5224	0.00048	−0.3412	0.00002	-
YCL036W	GFD2	−0.3546	0.00005	−0.4342	0.00023	−0.3447	0.00037	-
YDR360W	OPI7	−0.4144	0.00001	−0.3920	0.00002	−0.3042	0.00000	-
YDR209C	-	−0.3699	0.00002	−0.3455	0.00001	−0.3306	0.00000	-
YDL187C	-	−0.4446	0.00048	−0.3041	0.00013	−0.4404	0.00030	-

^a^ 1, 2, and 3 indicate three independent replicates. ^b^ “-” indicates unknown.

## Data Availability

The data presented in this study are available on request from the corresponding author.

## Supplementary Materials

The following are available online at https://www.mdpi.com/article/10.3390/cells10040888/s1, Figure S1: Sensitivity of *S. cerevisiae* to LiPF_6_, LiCl, and NaPF_6_, Figure S2: Photographs of plates in the absence or presence of 3 mM LiPF_6_, Figure S3: Growth comparison of BY4741 in YPD or YPG media, Figure S4: Mitochondrial morphology was affected by LiPF_6_, Figure S5: Detection of Cox14, Cox12, Qcr2, and Qcr6 protein expression changes, Figure S6: Mitochondrial F1F0 ATP synthase-related gene deletion strains show increased sensitivity to LiPF_6_, Table S1: Primers for identification of complementation strains, Table S2: Location distribution of the 75 genes associated with LiPF_6_-sensitivity.
