# Supplementary material for: A Genome-Wide Screen in Saccharomyces cerevisiae Reveals a Critical Role for Oxidative Phosphorylation in Cellular Tolerance to Lithium Hexafluorophosphate
Source: Cells. 2021 Apr 13;10(4):888. doi: 10.3390/cells10040888 (PMC8070311; doi:10.3390/cells10040888)
Supplement: Supplementary file 1 [file cells-10-00888-s001.pdf]

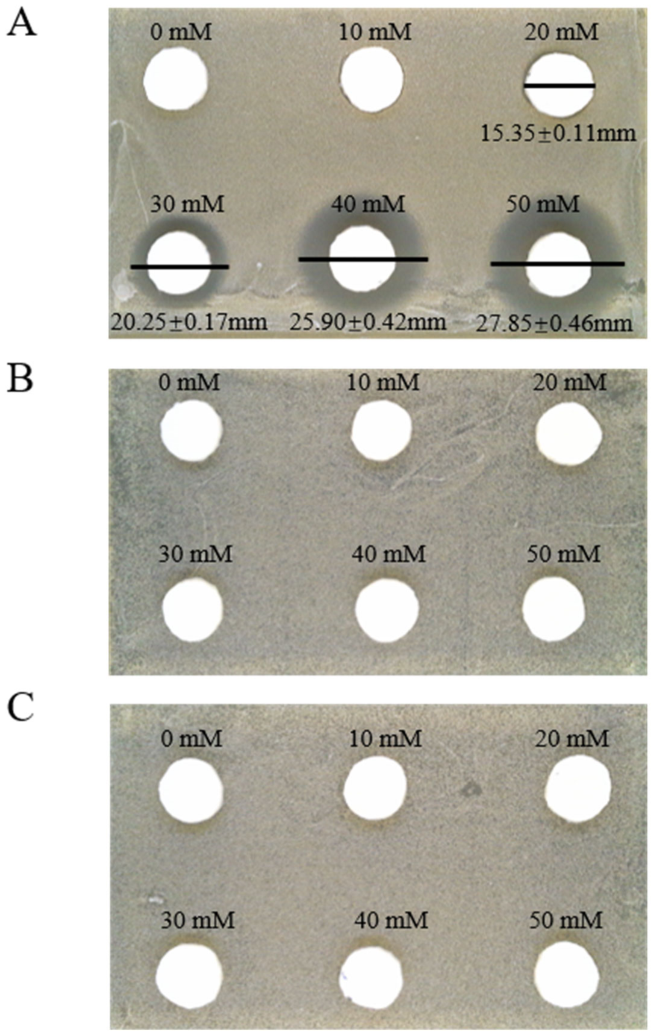

Figure S1 Sensitivity of *S. cerevisiae* to $LiPF_6$, LiCl, and $NaPF_6$.

The inhibition zone of yeast to different concentrations of $LiPF_6$ (A), LiCl (B), and $NaPF_6$ (C) were observed. The diameter of the growth inhibition zone was represented by means and error bars indicate SE.

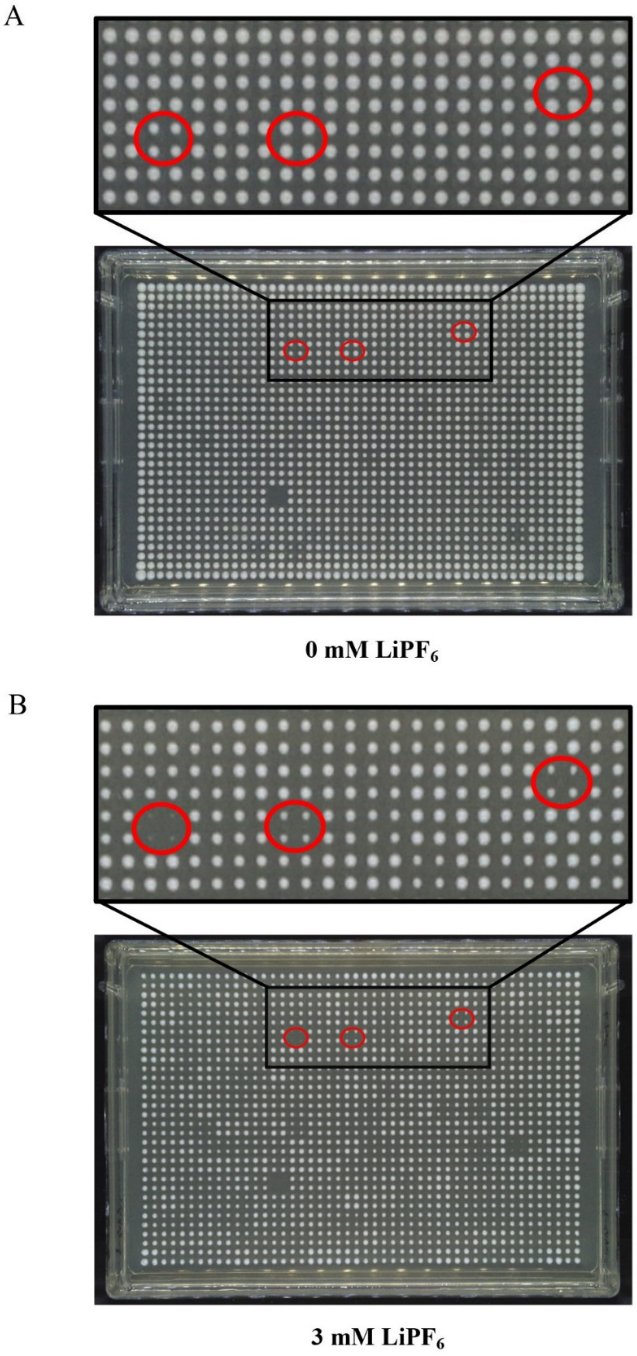

Figure S2. Photographs of plates in the absence or presence of 3 mM $LiPF_6$

A randomly selected plate was pinned onto solid YPD medium without (A) or with (B) 3 mM $LiPF_6$. The photograph depicts a set of 384 mutants, each mutant pinned four times to create a 1536-density array. Plates were photographed after 48 h incubation at 30°C. The small colonies marked with red circles are representative of $LiPF_6$-sensitive mutants. YPD, Yeast Peptone Dextrose.

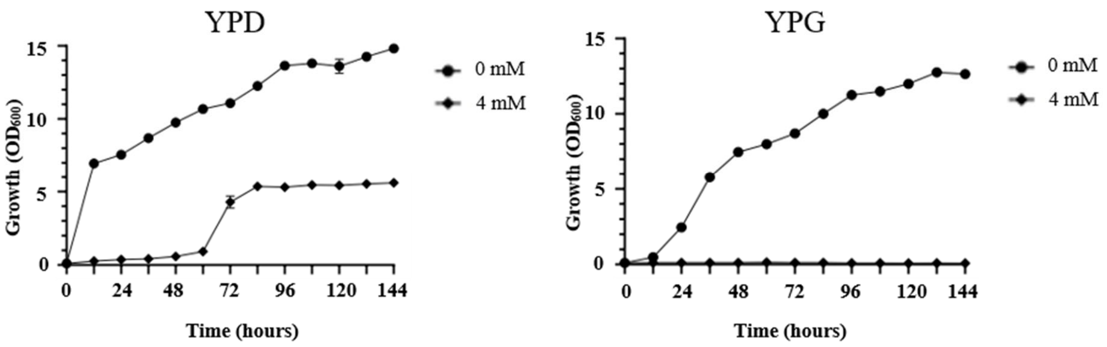

Figure S3 Growth comparison of BY4741 in YPD or YPG media

BY4741 was grown in liquid media supplemented with 2% dextrose (YPD) or with 3% glycerol (YPG) for 144 h at 30 °C in the absence or presence of $LiPF_6$. Growth curves were performed in triplicate. Growth was represented by mean OD_{600} values and error bars indicate SE.

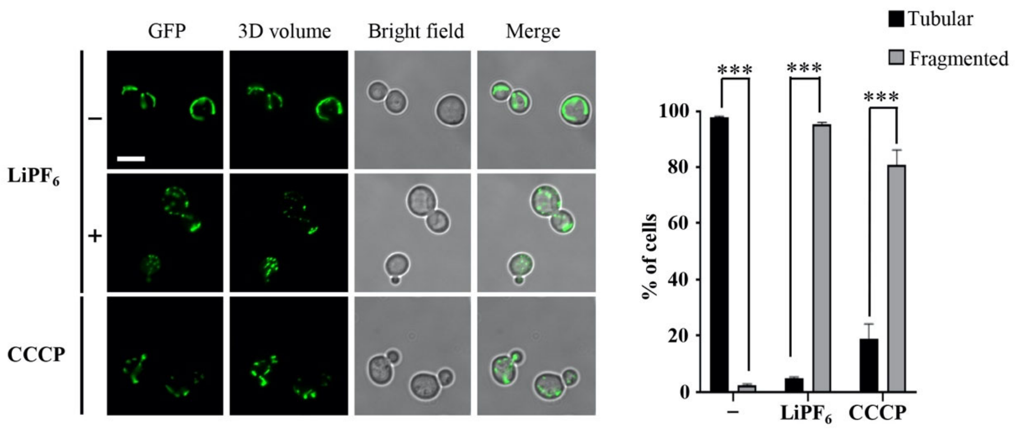

Figure S4 Mitochondrial morphology was affected by $LiPF_6$

Mitochondrial morphology of BY4741 cells expressing Ilv3–GFP was observed with or without $LiPF_6$. 10 μM CCCP-treated cells served as positive control. Cells were grown to saturation and diluted to an OD_{600} of 0.1 in SC-His. Cells were grown to an OD_{600} of 0.5 before treated with 0 or 0.5 mM $LiPF_6$. After 15 min culture, cells were collected and washed in phosphate buffered saline (PBS) two times before observation using a Zeiss Axio Observer 7 with Z stacks. Ilv3-GFP signals are shown on the left; 3D volume images of mitochondria are shown in the second column; bright field micrographs are shown in the third column; merged images are shown on the right. "−": without $LiPF_6$; "+": with $LiPF_6$. Bars, 5 μm. The percentage of cells exhibiting tubular or fragmented mitochondria was calculated. At least 200 cells of each sample were used for quantitation. Error values indicate SE from three independent experiments. CCCP, Carbonyl cyanide m-chlorophenylhydrazone.

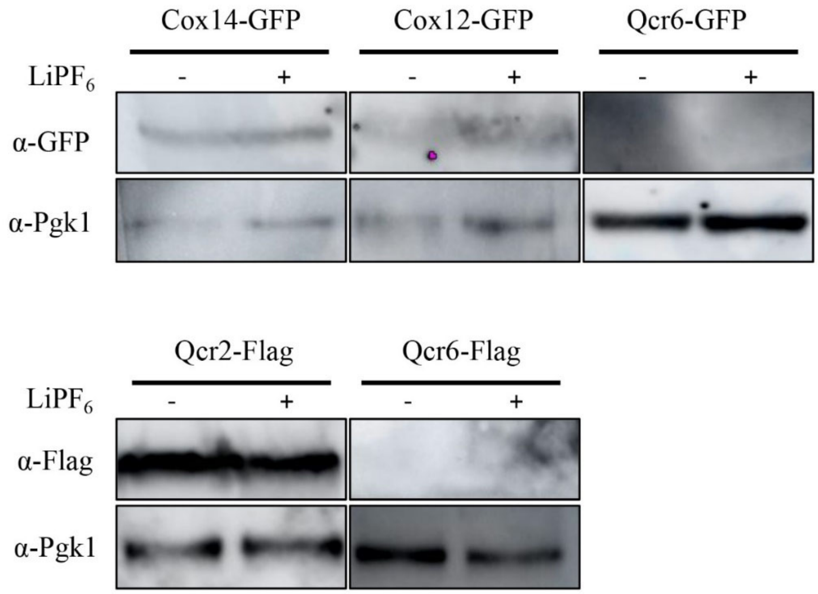

Figure S5. Detection of Cox14, Cox12, Qcr2, and Qcr6 protein expression changes

Western blot analysis of Cox14, Cox12, Qcr2, and Qcr6 protein expression before and after $LiPF_6$ treatment. Pgk1 served as a loading control. Proteins were fused with a GFP tag or a Flag tag. For Cox14, Cox12, and Qcr2, there were no significant differences between protein expression before and after $LiPF_6$ treatment. The expression of Qcr6-GFP and Qcr6-Flag could not be detected.

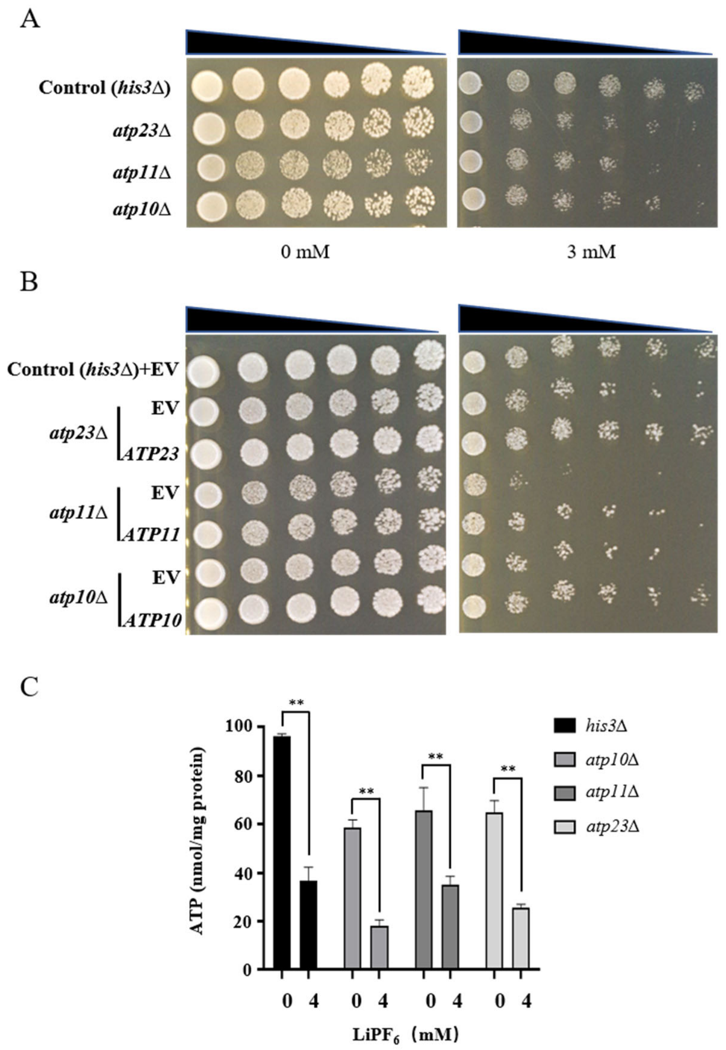

Figure S6 Mitochondrial F1F0 ATP synthase-related gene deletion strains show increased sensitivity to $LiPF_6$

(A) Spot test to verify screening results. The control strain (*his3△*), and *atp23△*, *atp11△*, and *atp10△* strains were grown to mid-log phase in YPD medium and then diluted to an OD_{600} of 0.5. Cells were serially diluted onto YPD agar plates either containing 3 mM $LiPF_6$ or no $LiPF_6$. Plates were photographed after 48 h of incubation at 30°C. Images shown are representative of triplicates. (B) Spot test of complementation strains. Deletion mutant strains transformed with empty vector or plasmid expressing the corresponding genes were grown to mid-log phase in YPD medium before diluting to an OD_{600} of 0.5 and cells were serially diluted onto YPD plates with 3 mM $LiPF_6$ or without $LiPF_6$. The control strain transformed with empty vector served as a control. (C) Detection of mitochondrial ATP synthetic activity of F1F0 ATP synthase-related

deletion mutants. The vertical axis represents the ATP content per mg protein. Error bars indicate SE from three independent experiments. **, $p < 0.01$, Student's t-test. SE, Standard Error.

Table S1 Primers for identification of complementation strains

Gene name	Primer pair	Primer sequence (5'-3')	Position
COX5A	Chl-F	GCAAGATGTGGCGTGTTAC	Corresponding to CmR 363-381 nt
	COX5A-R	CAGGCTCAGTAAGCTGTG	Reverse complementation with COX5A 185-202 nt
COX12	Chl-F	GCAAGATGTGGCGTGTTAC	Corresponding to CmR 363-381 nt
	COX12-R	CCACATAAGATTGCCAACAATG	Reverse complementation with COX12 76-97 nt
COX14	Chl-F	GCAAGATGTGGCGTGTTAC	Corresponding to CmR 363-381 nt
	COX14-R	CGTACTTCTTACCGTTCATG	Reverse complementation with COX14 114-133 nt
QCR2	Chl-F	GCAAGATGTGGCGTGTTAC	Corresponding to CmR 363-381 nt
	QCR2-R	CATAACGAGACCCTCCATG	Reverse complementation with QCR2 106-124 nt
QCR6	Chl-F	GCAAGATGTGGCGTGTTAC	Corresponding to CmR 363-381 nt
	QCR6-R	CCTTTTCTTCATGCTGCTCG	Reverse complementation with QCR6 90-109 nt

CmR: Chloramphenicol resistant gene on plasmid

Table S2 Location distribution of the 75 genes associated with $LiPF_6$-sensitivity

Location	Number	Percentage (%)
mitochondrion	19	25.3
cytoplasm	12	16.0
cytoplasm, nucleus	7	9.3
nucleus	5	6.7
punctate composite	2	2.7
ambiguous	2	2.7
ER	2	2.7
nucleolus, nucleus	1	1.3
nucleolus	1	1.3
endosome	1	1.3
cytoplasm, late Golgi	1	1.3
vacuole	1	1.3
actin	1	1.3
punctate composite, actin	1	1.3
punctate composite, endosome	1	1.3
Unknown	18	24.0
